# The Do-Well study: protocol for a randomised controlled trial, economic and qualitative process evaluations of domiciliary welfare rights advice for socio-economically disadvantaged older people recruited via primary health care

**DOI:** 10.1186/1471-2458-12-382

**Published:** 2012-05-28

**Authors:** Catherine Haighton, Suzanne Moffatt, Denise Howel, Elaine McColl, Eugene Milne, Mark Deverill, Greg Rubin, Terry Aspray, Martin White

**Affiliations:** 1 Institute of Health and Society, Newcastle University, Baddiley Clark Building, Richardson Road, Newcastle, NE2 4AX, UK; 2 Newcastle Clinical Trials Unit, Institute of Health and Society, William Leech Building, Faculty of Medical Sciences, Newcastle University, Newcastle, NE2 4HH, UK; 3 Institute for Ageing & Health, Campus for Ageing and Vitality, Newcastle University, Newcastle, NE4 5PL, UK; 4 School of Medicine and Health, Durham University, Queen's Campus, University Boulevard, Stockton-on-Tees, TS17 6BH, UK

**Keywords:** Randomised Controlled Trial, Welfare Rights Advice, Older People, Primary Care

## Abstract

**Background:**

Older people in poor health are more likely to need extra money, aids and adaptations to allow them to remain independent and cope with ill health, yet in the UK many do not claim the welfare benefits to which they are entitled. Welfare rights advice interventions lead to greater welfare income, but have not been rigorously evaluated for health benefits. This study will evaluate the effects on health and well-being of a domiciliary welfare rights advice service provided by local government or voluntary organisations in North East England for independent living, socio-economically disadvantaged older people (aged ≥60 yrs), recruited from general (primary care) practices.

**Methods/Design:**

The study is a pragmatic, individually randomised, single blinded, wait-list controlled trial of welfare rights advice versus usual care, with embedded economic and qualitative process evaluations. The qualitative study will examine whether the intervention is delivered as intended; explore responses to the intervention and examine reasons for the trial findings; and explore the potential for translation of the intervention into routine policy and practice. The primary outcome is the effect on health-related quality of life, measured using the CASP 19 questionnaire. Volunteer men and women aged ≥60 years (1/household) will be identified from general practice patient registers. Patients in nursing homes or hospitals at the time of recruitment will be excluded. General practice populations will be recruited from disadvantaged areas of North East England, including urban, rural and semi-rural areas, with no previous access to targeted welfare rights advice services delivered to primary care patients. A minimum of 750 participants will be randomised to intervention and control arms in a 1:1 ratio.

**Discussion:**

Achieving a trial design that is both ethical and acceptable to potential participants, required methodological compromises. The choice of follow-up length required a trade-off between sufficient time to demonstrate health impact and the need to allow the control group access to the intervention as early as possible. The study will have implications for fundamental understanding of social inequalities and how to tackle them, and provides a model for similar evaluations of health-orientated social interventions. If the health benefits of this intervention are proven, targeted welfare rights advice services should be extended to ensure widespread provision for older people and other vulnerable groups.

**Current Controlled Trials ISRCTN Number:**

ISRCTN37380518

## Background

### Socio-economic inequalities in health, income and older people

Health inequalities are universal across nations, societies and the human lifecourse [1-3]. Socio-economic differences in health persist into old age and social inequalities in self-reported physical and mental health widen in early old age [4]. The poorest older people have inadequate access to services essential to health and well-being [5]. Older people, especially those in poor health, are more likely to require additional income and support, including payments for care, domestic help and aids and adaptations to the home [6-8].

### Resource-based interventions to promote health

The vast body of evidence on socio-economic inequalities in health suggests a close relationship between access to resources and health status. Increasing an individual's or group's access to material, social or financial resources should result in improved health [9,10], yet little research has directly evaluated the impact of increasing resources on health [11]. A systematic review of ten North American randomised controlled trials of income supplementation experiments targeting a range of age groups, carried out in the late 1960s and 1970s, showed that none had reliably assessed the effects of increased income on health. The authors pointed out that, although such experiments are unlikely to be repeated, one way of assessing the health impact of increasing financial resources on health lies with assisting claimants to obtain full welfare benefit entitlements [11]. Tackling health inequalities has become a major policy priority for the UK government, highlighted, for example, in the white paper on public health (‘Saving Lives: Our Healthier Nation’). Following publication of the ‘Acheson Report’ [2] and the advent of Health Action Zones (HAZs) in the late 1990s [12], there was an increase in welfare rights advice projects linked to primary care in the UK. A voluntary organisation estimated in 2000 that there were >130 welfare rights advice services targeting primary care patients in England [13]. In 1999, 'Reducing Health Inequalities: an Action Report' [14], highlighted welfare rights advice as a potentially effective intervention to reduce health inequalities. This proposal was endorsed by the UK Government’s ‘Marmot Review’ in 2012 [3].

### Social welfare for older people and under-claiming of entitlement

In the UK, large amounts of social welfare benefits go unclaimed [15,16], and a disproportionate amount of these are the health-related benefit entitlements of vulnerable groups, such as older people [17,18]. Failure to claim entitlements is linked to a number of factors including the complexity of the benefits system [19], lack of knowledge about entitlements and difficulty in making claims [8,20-22]. In addition to the state pension, there are a number of means-tested and non-means-tested benefits that can be awarded if entitlement conditions are fulfilled. The level of under-claiming varies depending on the benefit concerned, but is estimated to be at least 30 % for the main benefits [23]. Entitlement to one benefit can often act as a ‘passport’ to others, since many of the benefits aimed at people over national retirement age are linked together in a complex network of entitlements that are often difficult for people to access without expert assistance.

### Welfare rights advice services and their evaluation

Our systematic review of the health, social and economic impacts of welfare rights advice services in healthcare setting [21], identified numerous studies that have demonstrated the financial and material benefits of such services. Welfare rights advice provided by local authorities, charities and voluntary organisations is known to increase uptake of benefits, particularly where this involves ‘active assistance’ with benefit claims [2,17]. Studies have also shown that receipt of benefit entitlements can be increased by providing information and advice in general practice, particularly in relation to those benefits that are health-related [24-28]. However, our systematic review only identified two studies that have investigated the health impact of welfare rights advice; [29-31] one of which found an improvement in health-related quality of life in some of the subscales of the SF36 [29,30]. However, both studies demonstrate the difficulties of identifying and measuring appropriate health outcome measures when assessing the health effects of welfare rights advice in primary care [29-31]. Neither study used a randomised controlled design, and both suffered from significant methodological weaknesses that render them inconclusive. Qualitative studies exploring the impact of welfare rights advice on clients in primary care identified a range of health-related outcomes that can potentially result from receipt of welfare rights advice, including changes in physical, behavioural and, in particular, psycho-social domains of health [8,20-22].

We conducted a pilot randomised controlled trial (RCT) to prepare for the definitive RCT described here, evaluating the impact of a domiciliary welfare rights advice service offered to people aged ≥60 years, identified via primary care in disadvantaged areas [7,8,32]. This pilot trial increased uptake of financial (median gain £55/week) and non-financial benefits (e.g. aids and adaptations to the home) in 58 % of participants [7], confirming the feasibility and success of the intervention from the point of view of accessing unclaimed benefits. It also provided vital information on the feasibility of such a trial, which has helped in planning the current definitive RCT.

Welfare rights (WR) advice interventions lead to greater income, but have not been rigorously evaluated for health benefits, in part because such research has previously been deemed unethical [33]. We present the protocol for a definitive RCT of WR Advice for people aged ≥60 years, and discuss the methodological and ethical issues that needed to be taken into account in designing the trial. We identified challenges relating to: randomisation, contamination, equipoise and control condition, length of follow-up, selection bias, outcome measures, generalisability and nature of the intervention. These are analysed in relation to scientific and ethical considerations.

## Objectives

The proposed RCT with embedded economic and qualitative process evaluations aims to answer the following questions:

1. What are the effects on health-related quality of life of a domiciliary welfare rights advice service targeting independent-living, socio-economically disadvantaged older people (aged ≥60 yrs) identified via primary care?

2. What are the cost consequences and what is the cost-effectiveness of a domiciliary welfare rights advice service targeting independent-living older people (aged ≥60 yrs) identified via primary care?

3. What is the acceptability to trial participants and relevant professionals of a domiciliary welfare rights advice service targeting independent-living older people (aged ≥60 yrs) identified via primary care?

4. What are the unanticipated consequences (positive or negative) of a domiciliary welfare rights advice service targeting independent-living older people (aged ≥60 yrs) identified via primary care?

### Study Design

**Figure 1:**
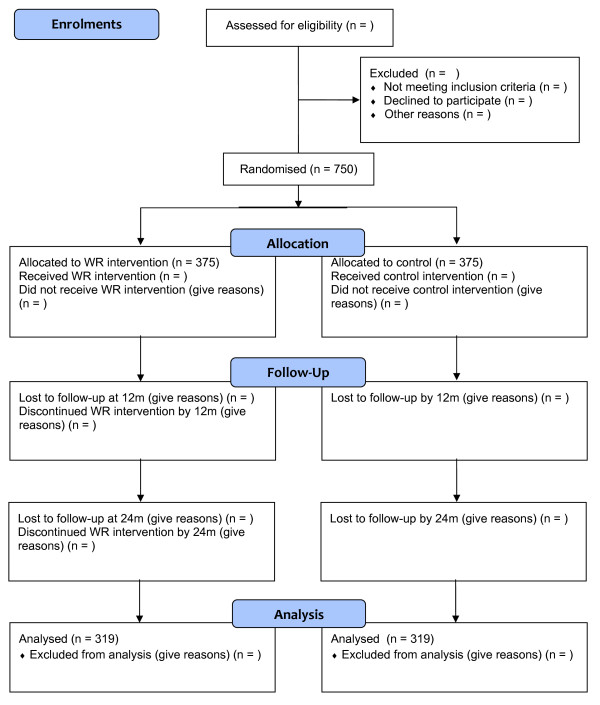
CONSORT 2010 standard RCT flow diagram for Do-Well study (numbers, where they appear, are estimates at this stage).

The study is a pragmatic, individually randomised, single blinded, wait-list controlled trial of welfare rights advice versus usual care, with embedded economic and qualitative process evaluations. The qualitative study will examine whether the intervention is delivered as intended, explore responses to the intervention and examine reasons for the trial findings, and explore feasibility of the translation of the intervention into routine policy and practice. The trial design is illustrated in Figure 1, which has been drawn according to the CONSORT guidelines [34].

### Primary outcome measure

The primary outcome is quality of life, measured using the CASP 19 questionnaire which was developed for use with older people [35,36]. CASP stands for the four domains of Control, Autonomy, Self realization and Pleasure. CASP 19 will be administered by face-to-face interview at baseline (pre-randomisation) and at follow-up 24 months post-randomisation, and by postal questionnaire at 12 months post-randomisation.

### Secondary outcome measures

The following secondary outcomes, based on the findings of previous research, including our own systematic review, qualitative study and pilot trial [7,8,20-22,32,37] will be collected:

· *Health status*, measured by the EuroQoL (EQ5D) [38]

· *Functional ability* measured by the modified Townsend activities of daily living scale [39]

· *Independence* categorised as: living independently or with carer support, in own home, with relations, care home or hospital (the latter two categories at follow-up only)

· *Mental health* measured by the PHQ-9 depression questionnaire [40-42]

· *Health-related behaviours* assessed by self-report to measure change in key indicator behaviours, such as smoking, alcohol consumption, diet (consumption of key food groups) and physical activity, as in our pilot RCT [7]

· *Mortality* assessed by identifying deaths at 12 months and 24 months from GP records (we will do this prior to commencing follow-up assessments, so as not to attempt to contact the recently deceased, which may cause distress to bereaved relations)

· *Social support and participation* measured by the Social Support Questionnaire [43].

· *Perceived financial wellbeing* measured by the Affordability Index [44].

· *Fuel poverty* measured where a household can achieve temperatures needed to maintain health and comfort for expenditure of less than 10 % of income [45]

· *Financial status* measured by a standard assessment tool developed and used in our pilot RCT [7]. This includes data on all sources of household income, including benefits, major outgoings (rent/mortgage, fuel bills etc.), debts and capital assets (i.e. home and savings). As well as these data, at follow-up detailed data will be collected (by WR Advisors) on new benefits received since baseline, including one-off (lump sum) payments and regular, weekly or monthly income.

· *Material (dis)advantage* measured through standard questions to ascertain home ownership, size of home (number of ‘living’ rooms), car ownership, and access to household amenities (such as central heating, cooker, fridge, freezer, etc.).

These outcomes will be assessed by structured, face-to-face interview at baseline (pre-randomisation) and 24 months post-randomisation.

### Other quantitative data to be collected

As well as demographic factors including age, sex, ethnicity, marital status and living arrangements, including dependants, data will be collected to assess the costs of the intervention, from public sector and treasury perspectives (see ‘Health economic analyses’ below). The service costs of delivering the intervention will be assessed by collecting data on staff salaries from all participating services, as well as data on typical caseloads. WR Advisors routinely record information on visits to clients and we will use these data to estimate time spent with study clients, as well as travel costs. These data will be used to derive an average cost per case of delivering the intervention to our intervention group participants, as in our pilot RCT [7]. To assess the treasury perspective, total gains in financial benefits for all intervention clients will be provided by WR Advisors. We will also estimate the cost to the treasury of non-financial benefits based on details of successful claims provided by WR Advisors. These costs will then be summed to derive average costs to the treasury per case for all intervention participants.

### Study population

#### Randomised controlled trial

##### Inclusion criteria – general practices

General practice populations in disadvantaged areas of North East England, including urban, rural and semi-rural areas, with no previous access to targeted welfare rights advice services delivered to primary care patients, will be included. All practices from participating social service districts will be ranked according to deprivation score (2010 English Index of Multiple Deprivation calculated at Middle Super Output Area level for practice postcodes, according to the method of Griffin et al [46].). Those practices in the lower two fifths of the deprivation ranking distribution without existing dedicated or targeted welfare rights advice services will be eligible for inclusion.

##### Inclusion criteria - patients

· Volunteer men and women registered with a general practice in one of 10 social services areas (1 individual per household)

· Aged ≥60 years

· Providing informed consent

##### Exclusion criteria - patients

· Resident in social care (residential) or nursing homes or hospitals at the time of identification and recruitment

· Diagnosed with terminal illness

· Cannot participate in the research by virtue of current physical/mental health

· Lack of fluency in written and spoken English

#### Qualitative study

A range of professionals involved in service commissioning, policy and strategy will be interviewed including: (i) public health/NHS (GP commissioning consortia); (ii) social and welfare rights services; (iii) Pension Service, Department for Work and Pensions; and (iv) the voluntary sector. Trial participants will also be purposively selected to take part in interviews (see below).

### Screening, recruitment, consent and randomisation

#### Identification and screening of trial participants

Practices will be recruited with the help of the Northern and Yorkshire Primary Care Research Network (PCRN-NY). The study will take place in 10 local authority districts (Stockton, Darlington, Middlesbrough, County Durham, Sunderland, South Tyneside, North Tyneside, Newcastle, Gateshead, Northumberland) which have agreed to provide welfare rights advice services. We plan to recruit two general practices per local authority district. Potentially eligible practices will be identified as described above. We will then liaise with Welfare Rights Services to establish whether any of these practices have existing dedicated or targeted welfare rights advice services, since this will render them ineligible. Next, we will ask the PCRN-NY to identify which of the practices still eligible have indicated willingness to participate in research. If more than two general practices from each list have expressed willingness to participate in research, we will order the remaining practices randomly and then contact them sequentially until two practices from each social services district have agreed to participate in the trial.

General practices in North East England have access (via PCRN-NY and the Comprehensive Local Research Networks (CLRNs)) to personnel and financial resources to identify and approach research study participants. Using PCRN-NY and CLRN personnel and financial resources, each participating practice will be asked to generate a random sample of up to 300 people aged ≥60 from their practice register. Practice staff will scrutinise their list to identify any patients terminally ill and patients known to be resident in hospital or long-term care, who will be excluded. They will also check to ensure that only one person per household has been selected for this list. If 2 or more people from the same address are found, one will be selected at random to be retained and the other(s) removed from the sample list.

#### Recruitment

This list of up to 300 names per practice will be randomly ordered and the first 100 patients on the list will be sent a letter and study information sheet by their GP, inviting participation in the trial. The letter will explain that, unless the participant objects (by returning an opt-out form to the practice within 2 weeks), their name and contact details will be passed to the research team, who will then contact them directly to discuss the trial further and seek informed consent. Patients’ views have been incorporated in the design of patient information sheets and consent forms and the opt-out approach has been approved by the Research Ethics Committee.

After two weeks, contact details of those who have not opted out will be passed to the research team. Research staff will contact these individuals and, if acceptable, arrange a face-to-face meeting at a mutually convenient time in the participant’s own home or another location of the participant’s choosing.

Our target for recruitment from each practice will be predetermined (depending on the final number of practices involved) in order to achieve the total sample. If this number is not achieved from the first 100 mailed, subsequent mailings of further names from the list of up to 300 will take place until the required number have been recruited. The number to be included in subsequent mailings will be determined by the number of responses already received (i.e. the yield rate of each wave of invitations). Since recruitment interviews will be spread over a six month period, this iterative recruitment process should not delay overall recruitment.

#### Consent

At the initial appointment, the research interviewer will first seek written, informed consent and then, if appropriate, proceed to collect baseline data. Interviewers will communicate in English, and if English is not the first language of any participant and (s)he is unable to speak it fluently, the participant will be excluded from the study. Friends, relations or carers will not be used as interpreters and interpreting services available to WR Advisors from local authorities will not be available for research interviews (the CASP19 has not been translated to other appropriate languages nor cross-culturally validated).

The researcher will assess if an individual has the capacity to consent. If it is established that an individual is unable to provide written consent because of literacy, vision or motor problems, it will be arranged for verbal consent to be taken in the presence of an independent witness (e.g. family member) who will initial, sign and date the consent form on the participant’s behalf.

Although unlikely to be a frequent occurrence, it is conceivable that a participant may lose mental capacity *during* the follow up period. We will explain to participants at baseline that, if this happens, we will retain the information that we gathered prior to his/her loss of capacity. The investigator and research interviewers will undertake all reasonable steps to protect the study participant. In accordance with the Mental Capacity Act 2005, nothing will be done to the person to which he or she appears to object (whether by showing signs of resistance or otherwise) except where what is being done is intended to protect him or her from harm or to reduce or prevent pain or discomfort.

#### Identification and recruitment of participants for qualitative sub-study

Interviews with approximately 30 purposively sampled trial participants will take place between 8–11 months and between 20–23 months from baseline (approximately 15 interviews in each period). Trial participants will be identified through the trial database and recruited to achieve a maximum variation sample with respect to group allocation, gender, age, benefit entitlements and any unanticipated consequences of the intervention identified at 12 month follow-up.

A sample of approximately 10 professionals/stakeholders will also be interviewed at 20–23 months from baseline. Stakeholders will include representatives of the Department for Work and Pensions, Benefits Agency, adult social services managers, welfare rights advisors, General Practitioners (GPs), primary care commissioners and directors of public health.

Trial participants will be asked during baseline assessment and consent procedures if they would be willing to participate in the qualitative interviews. Those selected for interview (trial participants and professionals/stakeholders) will be sent a letter of invitation and additional participant information sheet by the research team. Contact details for the researchers will be provided so that those approached to participate can ask any questions they may have before coming to a decision on participation. Separate informed consent will be taken for the interviews and lack of consent to participate in this element of the research will not prevent trial participants from continuing in the trial.

Sampling and interviews with both groups will continue until data saturation is achieved [47]. Interviews with trial participants will explore: acceptability of the intervention and research design; unanticipated consequences of the intervention; and perceived impacts of the intervention. Interviews with stakeholders will explore: acceptability of the intervention, training and research; fidelity of the intervention; and likely implications of the intervention for translation into routine policy and practice, both within the North East and more widely.

### Sample size

#### Trial sample size and power

A minimum of 750 participants will be randomised to intervention and control arms, providing 90 % power at 5 % significance level to detect a 1.5 unit difference in mean CASP19 score [35,36] between intervention and control groups, assuming a standard deviation of 8.7 and a correlation between baseline and 24 months of 0.74 [48], and 15 % attrition over 24 months (as experienced in our pilot RCT) [7]. There has been no published work to establish a meaningful or clinically important difference on the CASP19 scale. However, we have used data from two waves of the English Longitudinal Study of Ageing in those aged ≥60 yrs to investigate the adjusted mean difference in CASP19 at Wave 2 between groups whose social or health circumstances had changed [48].

Examples of changes in CASP19 score associated with changes in health or social circumstances that we might expect to see in the proposed trial include: ‘developed limiting illness’ -2.8 units; ‘developed depression’ -2.7units; ‘lost access to car’ -1.8 units; ‘increased chance will not meet financial needs’ -1.1 units. These differences on the CASP19 scale suggest that a difference of 1.5 units would represent a ‘clinically’ important difference.

The chosen sample size should also provide power to demonstrate some clinically significant differences in secondary outcomes. For example, 750 participants will provide 90 % power to detect a difference between a prevalence of 11 % and 4 % of clinically significant depressive symptoms (PHQ-9 score ≥10).

#### Qualitative sub-study sample size

Sample size for the qualitative sub-study will be determined by data saturation. We anticipate that up to 30 trial participants and up to 10 stakeholders will be included.

### Study intervention details

#### Intervention

Welfare rights advice consultations and active assistance with benefit claims will be offered and delivered in participants’ own homes, tailored to individual needs by a trained WR Advisor employed by a local authority or Citizens’ Advice Bureaux (CAB) in North East England. Following randomisation, intervention group participants will be given an appointment in their own home with a WR Advisor within 2 weeks, during which participants will undergo a full benefit entitlement assessment involving: assessment of financial, material and welfare status; assessment of previous benefit entitlement and claims; discussion of current entitlement and options for action, including new claims (financial and non-financial). Active assistance with benefit claims and other welfare issues will be given. Complex claims or those referred for further assessment or tribunal will be managed in the usual way by WR Advisors. Participants will be followed up intermittently by WR Advisors until they no longer require assistance (cases are usually ‘closed’ once all claims and appeals have been resolved satisfactorily). It is expected that approximately 50 per cent of claims will be resolved within 3 months, but some may take up to 12 months [7]. The intervention will be funded and provided by WR Advice departments and CAB in 10 local authority areas across the North East by trained WR Advisors.

The North East Strategic Health Authority has provided funding for training of WR Advisors to ensure a consistent approach to delivery of the intervention. Such training was delivered in the context of our pilot RCT [7,8] and Newcastle City Council, Welfare Rights Service has agreed to provide similar training for this trial.

#### Comparator (wait-list control condition)

Participants randomised to the control group will receive ‘usual care’ from both health and social services after randomisation until they have completed their 24-month follow-up assessment. They will be given no advice regarding welfare rights as a part of the study intervention during this period. However, they may independently seek welfare rights advice from the local authority, CAB or voluntary sector organisations. If this occurs, they will remain in the trial, but details of such advice and ensuing claims and outcomes will be recorded at the 24 months follow-up assessment. Following their 24-month follow-up assessment, they will receive the intervention, as delivered to the intervention group (described above), including all follow-up visits by WR Advisors and assistance with claims and appeals over the following months, until all claims have been resolved.

### Long-term care

Both intervention and control group participants will remain clients of the welfare advice service beyond the end of the trial, if necessary, until such time as their help is no longer needed, as per usual welfare rights advice service protocols.

### Randomisation

Following baseline measurements, participants will be randomised in a 1:1 ratio to intervention or control condition. Research interviewers will notify the project administrator after each baseline interview that a new participant has been successfully recruited. The administrator will hold sequential allocation tables for each practice, independently generated from random numbers prior to recruitment. The administrator will allocate all participants to intervention or control group in the sequence that they are recruited and immediately send each participant a standard letter informing them of their group allocation. The administrator will also immediately inform the appropriate WR Advisor of the contact details of each newly allocated intervention group participant and indicate that they are to be seen within 2 weeks. WR Advisors will be sent lists of control group participants to assess 24 months later. The research interviewers will not be notified of allocation status to ensure that they remain blinded for the duration of the study.

### Study data

#### Data handling and record keeping

Study data will be entered directly into a secure database during interviews for processing and management, and a record of any changes made to the data post-entry will be maintained. All personal information obtained for the study will be held securely at Newcastle University and will be treated as strictly confidential. Entry and verification of twelve month data from self-completion questionnaires will be outsourced to a data entry company.

 Data collection and transfer in this study will comply with UK NHS Research Ethics Service (NRES) http://www.nres.npsa.nhs.uk/ and Caldicott guidelines http://www.dh.gov.uk/en/Managingyourorganisation/Informationpolicy/Patientconfidentialityandcaldicottguardians/DH_4100563 and the Data Protection Act (1998) http://www.dh.gov.uk/en/Managingyourorganisation/Informationpolicy/Recordsmanagement/DH_4000489. All patients will be allocated a unique study identifier, which will be used on all data collection forms and questionnaires to preserve confidentiality; names or addresses will not appear on completed questionnaires or other data collection forms. Only a limited number of members of the research team will be able to link the unique identifier to patient-identifiable details (name, address and telephone number) which will be held on a password-protected database. All study documentation will be held in secure offices, not open to the public and all members of the research team with access to identifiable or anonymised data will operate to a signed code of confidentiality. Transmission of original or hard copy records (e.g. questionnaires, interview recordings) will be by secure fax, post or hand delivery by members of the research team or by the WR Advisors. Participants will be informed in the patient information sheet about the transfer of information to the research team and about levels of access to patient identifiable data, and will be asked to consent to this. Any data used in publications from the Do-Well study will be fully anonymised; it will not be possible to identify individual patients from such publications.

At the end of the study, original questionnaires, interview transcripts and consent forms will be securely archived for 15 years following publication of the last paper or report from the study, in line with Sponsor policy and Newcastle Clinical Trials Unit (NCTU) standard operating procedures. This will also allow any queries or concerns about the data, conduct or conclusions of the study to be resolved. Both sets of data will be archived after 5 years. Anonymised data will be submitted to a national archive collection.

#### Statistical analyses

Analyses of covariance and regression methods will compare primary and secondary outcomes between intervention and control groups at 24 months, adjusting for baseline outcome values and any imbalance in other covariates as appropriate.

Analyses will be by intention-to-treat. It will be necessary to consider any difference in attrition rates, and the non-randomness of the attrition, when comparing quality of life between the two groups. In the pilot RCT only 7/126 (5.5 %) died during the 24 month follow-up, so it is thought unlikely that methods for joint modelling of survival and longitudinal data will be necessary.

Exploratory sub-group analyses will also be undertaken, for example to examine differences in outcome between men and women, by age and by amount and type of benefits received.

#### Health economic analyses

The economic evaluation will consist of a cost analysis conducted from the perspectives of public sector services (‘Do-Well’ service delivery costs), and that of the Treasury (total cost of additional benefits paid out). The mean change in benefits and the mean change in total income of participants will also be calculated.

The cost analyses above will be used in conjunction with study outcomes to produce a cost consequences analysis [49]. If any significant change in EQ-5D health utility scores can be attributed to the intervention, we will undertake a cost-utility analysis [49]. The assumptions that underpin any such cost-utility analysis will be subjected to one-way sensitivity analysis [50] and, in addition, extensive probabilistic sensitivity analysis [50] will be used with results presented in the form of cost-effectiveness acceptability curves [50].

#### Qualitative analyses

All interviews will be digitally recorded (with permission) and transcribed verbatim. Data will analysed thematically following the Framework method [51] with constant comparison [52] and deviant case analysis [53] to enhance validity, supported by NVivo software [54].

## Ethics

A favourable ethical opinion has been received from the UK NHS National Research Ethics Service NRES Committee South West - Exeter (reference number: 11/SW/0260).

## Discussion

This is an innovative trial designed to assess whether welfare rights advice delivered in their own homes to people from disadvantaged areas aged ≥60 years leads to improvement in health-related quality of life. The study is built on extensive prior research, which has led to consideration of a range of ethical and methodological issues. These are discussed in detail below.

### Study design, level of randomisation, contamination and dilution

An appropriate trial design is required, preferably with both randomisation and concurrent controls. Individual level randomisation is preferable to cluster randomisation (e.g. at general practice level) as it requires a smaller sample size. The potential problem with individual level allocation is that there may be ‘contamination’ between intervention and control participants in the same general practice. Where welfare rights advice is available from an open access service delivered in the general practice this is more likely to be the case. However, by using a WR advisor who only sees patients in their own homes, we found in our pilot RCT that contamination did not occur; no control participants independently sought welfare rights advice during a follow-up period, albeit of only 6 months [7].

### Equipoise and the control condition

A key consideration in designing the proposed trial was whether there is genuine equipoise. Welfare rights advice is known to increase access to financial and material resources in eligible clients. However, our systematic review of published and grey literature indicated that there is, as yet, no conclusive evidence that welfare rights advice leads to positive or negative changes in health [21]. We discussed these findings with welfare rights advisors, with directors of adult social services, with a selection of GPs and with members of the public in our target age group. We found each of these groups to be in equipoise with regard to the proposed trial health outcomes. Having established this, we also carefully considered the issue of study design and the ideal and feasible control conditions.

Ideally, controls should be adults as similar as possible to intervention group participants, but should not receive welfare rights advice, nor claim or receive new benefits, during the period of outcome follow-up. In clinical trials, it is usual to withhold the intervention from the control group because the health benefits of the intervention are not proven (i.e. clinical equipoise exists). Whilst this is the case with regard to the health impacts of welfare rights advice, as indicated above there is adequate evidence that welfare rights advice leads to significant financial and material gains for a proportion of recipients. Thus, it is considered ethically problematic to identify that control group participants are eligible to receive additional financial benefits, but either to keep this information from them, or to tell them of their eligibility but not give them advice or help with claims. To circumvent this dilemma, we proposed that control participants should not receive a welfare rights assessment until the end of the trial period (i.e. following final outcome measurement). The full intervention (i.e. a full benefit assessment and active assistance with claims until resolved) will then be offered.

There may be concerns about whether the welfare rights advisors might feel tempted to offer benefits advice to controls before the two year ‘wait period’ has elapsed. This will not be possible for the simple reason that the welfare right advisors will not know the names of controls until a few weeks before their benefits assessment is due (i.e. two years after the their baseline assessment). Control participants’ names will be held securely by the study team over this period.

The proposed design thus avoids unfairly raising expectations among controls. It also helps to avoid the potential problem of contamination, which could arise if control participants independently sought welfare advice (leading to dilution of the outcome effect), although we will not make any attempt to prevent this. The control condition is therefore, in effect, a ‘wait-list’ control, whereby the control group will wait to receive the intervention 24 months after the intervention group.

It is, of course, possible that some members of both intervention and control groups may die during the proposed 24 months follow-up period, and we would expect this in the course of any prospective study of this age group. In our pilot study, we recorded 7 deaths (4 intervention group, 3 control group) after 24 months follow-up [7].

The proposed design of this RCT is fair because, at present, this kind of intervention is not routinely available to primary care patients and is generally only available to those who seek such services or are referred to them by a health or social care professional (e.g. a hospital social worker); these options remain open to patients in this trial. When targeted services are available in primary care, they tend to be short term and *ad hoc*. If we find any general practices in participating districts with access to such services they will be excluded from this trial. Genuine equipoise exists for the proposed health-related outcomes because participants will not be denied any entitlement that they would otherwise have received and, at present, the health impact of the proposed intervention is unknown [21].

### Pragmatic versus explanatory

Not all participants in the intervention group will be eligible for additional benefits, and for those who are, they may receive variable amounts of financial and non-financial benefits. Ideally, we would wish to examine the health impact of receiving versus not receiving such benefits, as well as examining the potential for a gradient of effect (‘dose-response’ relationship) by amount of benefit received. However, to do so would require a substantially larger sample size. In practice, therefore, the receipt of welfare rights advice is the intervention we are evaluating (rather than receipt of specific benefits), since ‘welfare rights advice’ is the service being delivered. The proposed trial is therefore a pragmatic (intention-to-treat) RCT of this complex intervention. Nevertheless, we will also assess the potential for exploratory sub-group analyses looking at differential effects by participant characteristics (such as age and sex), receipt/non-receipt of, and levels of any benefits received. We anticipate that the trial will therefore contribute both to answering the question of whether the complex welfare rights advice intervention is effective in improving health and to providing new evidence on the theoretical question of whether increasing resources leads to better health [9].

### Length of follow-up

To enable accurate assessment of the health and social effects of welfare rights advice, an appropriate length of follow up is required. Experience from previous work suggests that considerable time may elapse between first advice session and receipt of new financial or material benefits. Often this is between 3 and 6 months, but can be longer if the case is not straightforward or if there is an appeal. For example, in our pilot RCT, 45 % had received their entitlements by 3 months after their welfare assessment, 85 % after 6 months, 95 % after 9 months and 100 % by 12 months [7]. Given such delays in receipt of benefits, as well as the fact that once received they need to be spent, it seems unlikely that they will have substantial impacts on health within the first 12 months. In our pilot, which was not adequately powered for substantive analyses, we found no suggestion of differences in health-related outcomes between intervention and control groups after 6, 12 or 24 months (although controls received the intervention after 6 months) [7]. Nevertheless, it seems likely that the longer the delay between receipt of intervention and measurement of outcomes, the greater the chance of demonstrating a substantive effect on health.

To assess the acceptability of a range of delays in receiving the intervention among control group participants, we undertook an experiment in the context of a focus group discussion with a representative sample of low income, older people. To achieve this, simulations of the RCT randomisation procedures were undertaken. The first simulation concerned a typical drug trial and participants were given different coloured sweets depending on whether they were in the intervention or control groups. Then, randomisation for the proposed trial was simulated. The concept of equipoise, with regard to the health impacts of welfare rights advice was explained. Next, each group member was given an envelope from which they found out whether they were in the control group or the intervention group. If in the intervention group, they were allocated various types of benefit (e.g. Attendance Allowance + Council Tax Benefit + Housing Benefit) and the monetary value of these was revealed to them. We then talked through various time delays until the control group would also receive their welfare rights assessment and advice. The time delays used were 3 months, 6 months, 9 months, 12 months, 18 months, 24 months, 36 months and 60 months. The initial response to the design was that it was unfair on the control group. However, when it was explained that: (i) while such services exist, they are not routinely targeted at or delivered to all people aged over 60, but only available on referral or demand; (ii) the findings of this study could influence the development of such services, involving collaboration between health and social services; and (iii) that a substantial ‘wait’ between intervention and control groups is needed to establish any differences in health outcome, the consensus of the group was that a delay of 24 months would be acceptable in the context of the proposed trial. We have, therefore, opted for a wait-list design for the proposed RCT, with a 24 month follow-up period for the main outcome assessment, followed immediately by delivery of the full intervention to the control group.

### Selection bias

In our pilot RCT, GPs wrote to random samples of people aged over 60 years, inviting them to respond with an indication of their willingness to participate in the trial, i.e. to ‘opt in’ [7]. Using this method, 36 % initially agreed to participate, 14 % declined to participate and 50 % failed to respond. Low levels of positive response to an ‘opt in’ approach carry risks of participation bias [55,56]. In their work on evaluating the impact of welfare rights advice for the Department of Work and Pensions [36], Sainsbury and colleagues routinely use an ‘opt out’ method of recruitment for similar populations [57]. With the agreement of NRES we are using this type of recruitment method in this trial. This should significantly reduce the potential recruitment bias associated with ‘opt in’ recruitment methods and significantly increase the efficiency of trial recruitment.

### Choice of outcome measures

Previously reported studies of the health effects of welfare rights advice have restricted reported health outcomes to general measures of health or psycho-social functioning (such as the SF36 [29,30]) together with measurement of financial gains. In our earlier qualitative research among recipients of welfare rights advice, we identified a range of potential benefits of advice: [22]

*Health* (improvements in anxiety, depression, insomnia, reductions in medication or consultation, and health promoting changes in smoking, diet, physical activity, alcohol consumption)

*Social* (improvements in family or other relationships, increased ability to work, ability to care for relatives, etc.)

*Financial* (debt rescheduling and receipt of new benefits, e.g. Attendance Allowance, Disability Living Allowance, Mobility Allowance, Invalid Care Allowance, Incapacity Benefit, Housing Benefit, Income Support)

*Material* (e.g. access to free prescriptions, council tax exemption, entitlement to respite care, meals on wheels, re-housing or home modifications etc.)

The qualitative findings identified perceived benefits of the intervention in terms of:

increased affordability of necessities

increased capacity to manage unexpected future problems

decrease in stress related to financial worries

increased independence, including ability to travel, shop, visit GP etc.

increased ability to participate in family life and society

We undertook further qualitative work with study participants in our pilot RCT as well as collected planned outcome measures [8]. The pilot trial was not sufficiently powered for substantive analyses, but the feasibility of measurements was good, and well tolerated by older people.

These findings, together with those from recent, similar research [37] point to the most significant health-related impacts of welfare rights advice being on quality of life, independence, social participation and mental health. There is no single, ideal outcome measure that captures all of these domains, but the CASP19 instrument [35,36], developed specifically with a view to measuring quality of life in older people, comes close and has been recommended by Corden et al as a composite measure of the impact of welfare rights advice [36]. It is a self-reported summative index, comprising 19 Likert scale items in 4 domains: control, autonomy, self-realisation and pleasure [35]. Its performance has been examined in several prospective studies, including the English Longitudinal Study of Ageing (ELSA) [48].

### Generalisability

Our pilot trial was undertaken in one social services district (Newcastle upon Tyne) and in 4 general practice populations [7]. However, we know, from other work and discussions locally, that service delivery in welfare rights advice varies from area to area, as do general practice populations. To enhance the potential generalisability of the results, the RCT will therefore be undertaken across a range of geographical and local authority areas (including urban and rural) and general practices. It is possible, indeed likely, that the present welfare regime will change during the course of the trial. The proposed intervention is not dependent on any particular set of benefits and is adaptable to any new regime. This adds to its future generalisability.

### Target Population

Although we recognise that isolated older people who are eligible for benefits may live in all areas, in order to maximise the efficiency (and impact) of welfare rights advice services provided through primary health care, this RCT focuses on practice populations in socio-economically disadvantaged areas. Eligibility for health-related benefits (and failure to claim) increases with age, particularly post-retirement, although there are other key target groups such as single parents, non-claimants most likely to be accessed through primary care are predominantly in older age groups [15,16,22,37]. This trial therefore focuses on a predominantly post-retirement population (aged ≥60 years), residing in areas of economic deprivation.

### Nature of the intervention

The intervention to be delivered in the proposed trial is based on standard WR Advice services, of the type that can be found across local authorities in England. Conventionally, however, these services are available only on demand or by referral. Thus, for example, an older person admitted to hospital may be referred by a hospital social worker, doctor or nurse for benefits assessment prior to discharge. Only some services have undertaken targeting of WR Advice at a population level [21]. Those that have done so have found that there is a significant level of under-claiming in the general population and in particular among older people [18]. The proposed intervention is therefore a modification of a standard WR Advice service to target proactively a particularly vulnerable population in which we know there are high levels of under-claiming (i.e. over 60s in disadvantaged areas). The only reliable population registers in England at a local level are the primary care patient registration lists held by GPs and PCTs, which will be used to sample this target group selectively.

In our pilot RCT, we identified that efficiency and effectiveness (in terms of successful claims) could be maximised by making the service domiciliary, since a substantial proportion of over-60s have limited mobility and clients often need access during assessments to information kept at home [22,58]. Domiciliary visits also proved more popular with clients. We also found that welfare rights advisors need to provide ‘active assistance’ with claims, for example completing claim forms for clients, since this is a key barrier to claiming [8,20]. Lastly, GPs need to have appropriate awareness of welfare entitlements and, for health-related benefits in particular, an understanding of the medical criteria on which decisions are made so as to be able to support reasonable claims effectively in medical assessments requested by the benefits agency. Good communication between GPs and welfare rights advisors is essential to facilitate this. In our pilot RCT, we delivered education and training on these issues to all GPs in participating practices [7,8], another feature that is included in the proposed definitive RCT.

## Conclusions

We have presented the protocol for a definitive RCT of domiciliary welfare rights advice for socio-economically disadvantaged people aged ≥60 years recruited via primary health care, with embedded economic and qualitative process evaluations. While relatively straightforward, the design of the trial presented ethical and methodological challenges requiring a wait-list control group design. The study design is based on extensive prior research, including a systematic review, pilot RCT and qualitative studies [7,8,21]. Study recruitment will commence in April 2012 and the study is expected to report in May 2015. If the health benefits of this intervention are proven, targeted welfare rights advice services could be extended to ensure widespread provision for older people and other vulnerable groups, through collaboration between social services and primary care trusts or commissioners. The results of this trial may also have implications for the development of other resource-based interventions to tackle inequalities.

## Competing interests

The authors declare that they have no competing interests.

## Authors’ contributions

MW, SM and DH had the original idea for the study, were involved in the detailed design of the study, commented on successive drafts of the manuscript and approved the final version. CH was involved in the detailed design of the study, drafted the manuscript and approved the final version; EMc, MD, EM, GR and TA were involved in the detailed design of study, commented on successive drafts of the manuscript and approved the final version. MW is the Chief Investigator and acts as guarantor for the study. All authors read and approved the final manuscript.

## Pre-publication history

The pre-publication history for this paper can be accessed here:

http://www.biomedcentral.com/1471-2458/12/382/prepub
